# Exploring the Microbiome Analysis and Visualization Landscape

**DOI:** 10.3389/fbinf.2021.774631

**Published:** 2021-12-02

**Authors:** Jannes Peeters, Olivier Thas, Ziv Shkedy, Leyla Kodalci, Connie Musisi, Olajumoke Evangelina Owokotomo, Aleksandra Dyczko, Ibrahim Hamad, Jaco Vangronsveld, Markus Kleinewietfeld, Sofie Thijs, Jan Aerts

**Affiliations:** ^1^ CENSTAT, Data Science Institute (DSI), Hasselt University, Diepenbeek, Belgium; ^2^ VIB Laboratory of Translational Immunomodulation, VIB Center for Inflammation Research (IRC), Hasselt University, Diepenbeek, Belgium; ^3^ Department of Immunology and Infection, Biomedical Research Institute (BIOMED), Hasselt University, Diepenbeek, Belgium; ^4^ Center for Environmental Sciences, Environmental Biology, Hasselt University, Diepenbeek, Belgium; ^5^ Department of Plant Physiology and Biophysics, Faculty of Biology and Biotechnology, Maria Curie–Skłodowska University, Lublin, Poland

**Keywords:** microbiome, visual analytics, data visualization, bioinformatcs, data analysis, biostatistics

## Abstract

Research on the microbiome has boomed recently, which resulted in a wide range of tools, packages, and algorithms to analyze microbiome data. Here we investigate and map currently existing tools that can be used to perform visual analysis on the microbiome, and associate the including methods, visual representations and data features to the research objectives currently of interest in microbiome research. The analysis is based on a combination of a literature review and workshops including a group of domain experts. Both the reviewing process and workshops are based on domain characterization methods to facilitate communication and collaboration between researchers from different disciplines. We identify several research questions related to microbiomes, and describe how different analysis methods and visualizations help in tackling them.

## 1 Introduction

The human gut microbiome has been the topic of many academical studies over the latest years, as several diseases like multiple sclerosis and inflammatory bowel disease, have been found to be connected to it (Wilck et al., 2017; Allaband et al., 2019). Studies even suggest that there is a link between the gut microbiome and depression (Dash et al., 2015; Winter et al., 2018). Tripathi et al. (2018) noted that although much progress has been made in this research field, a framework of aggregated scientific knowledge about the topic (one needs to pose meaningful hypotheses) is still lacking. The authors therefore advocate for more discovery-driven, and tool-driven research projects instead of traditional, hypothesis-driven studies conducted using hypotheses-driven statistical or mathematical models. The reasoning behind this inductive approach, from which we start with a hypothesis-free exploration of the data, is that it can lead to unanticipated interesting questions as well as deeper insights of understanding. A promising and by now well-established technique to support hypothesis-free data exploration, are interactive data visualization and Visual Analytics (VA) (Van Wijk, 2005; Keim et al., 2010). Visualization experts play an important role in this as they possess the knowledge and visual literacy to perform visual analysis, and develop meaningful interactive data visualizations. Data visualization projects, and the interplay between visualization experts and domain experts therefore becomes more prominent in different research fields; e.g., social sciences (Lamqaddam et al., 2020), archaeology (Panagiotidou et al., 2020), and microbiome research. To work closely with domain experts, and performing a good requirement analysis is key for the visualization experts to succeed in the development of meaningful visualization tools (Knoll et al., 2020). This involves the visualization expert(s) to gain sufficient background knowledge in the research domain to understand expert’s needs, and domain experts to express their domain tasks, data types and analysis (Sakai and Aerts, 2015).

In this paper, we provide a picture of how (interactive) data visualization and visual analytics are currently used in microbiome research. To do so, literature covering visual analysis pipelines, visualization methods and visual analytic tools designed for microbiome research were reviewed and discussed in interactive expert panel focus groups. These interactive workshops were organized based on the principles of Kerzner et al. (2019) and Gray et al. (2010), using an informal setting in which discussion was facilitated through brainstorming games (e.g., Post-up, Card sort).

## 2 Materials and Methods

Data and material for the analysis was collected using a combination of literature review and collaborative workshops with a panel of experts related to microbiome research.

### 2.1 Literature Review

Literature was hand collected based on a google scholar search on “microbiome visualization,” “microbiome visual analysis,” and “microbiome studies interactive analysis.” To be as inclusive as possible, additional tools were added if referenced in one of the papers within this selection. Nevertheless, the final collection may not be exclusive. In total, 31 papers published between 2009 and 2021 were selected. This should give an accurate presentation of the analysis tools landscape. Note, that because of the special interest in the visual analytics aspect, a strong emphasis on visualization tools was laid in the search and collection process.

The review process was done manually. From each paper we extracted general information on the tool; such as the platform the tool is hosted on, the input formats of the data, and the aspects of the microbiome that could be revealed using the tool (e.g., diversity indices, differential relative abundances, etc.). In addition, we described which methods were used to extract information on the several microbiome aspects as well as the visualization method (if not overlapping) used for visual interpretation. Note that for the interest of this study, only analyses to perform on operational taxonomic unit (OTU) or amplicon sequence variant (ASV) tables were taken into account. This paper will not cover the process of transforming raw sequence data (.fastq files) into readable OTU/ASV tables.

### 2.2 Evaluation Methods

To analyze and draw conclusions of the observations, two techniques coming from the business environments were used to facilitate insight generation by revealing underlying patterns; being a *closed cart sorting* game (Sakai and Aerts, 2015) and the use of a *history map* (Gray et al., 2010). Both were conducted individually prior to the expert panel focus group discussions.

In *card sorting*, the objective is domain characterization, which is crucial in visual design. As visualization experts might not have sufficient background knowledge in the field of microbiome research, “*expert’s need*” have to be extracted in more abstract low-level tasks (Munzner, 2014). In this card sorting game, these abstractions were made based on the literature. The rules of the game are simple, a set of cards need to be sorted into meaningful categories. Cards can represent items, objects, pictures, names or attributes. In this case a closed Card Sort was conducted, meaning a set of predetermined categories is used; each category representing a feature (aspect) of the microbiome that could be identified in the analysis tools. The cards to be sorted contained the statistical methods, visualization algorithms and visual designs that were found in the same analysis tools to compute and represent these aspects. The sort in this exercise was based on the frequency of occurrence in literature (i.e., if PCoA was used to visualize between sample diversity, the “PCoA” card was assigned to the “between sample diversity” class). An example of how this was done can be found in Supplementary Figure S1 in the supplementary materials.

The *history map* (Gray et al., 2010) is used to familiarize new people with an organization’s culture and history during periods of rapid growth. The idea is to ask employees share memories about certain topics (e.g., company successes, changes in leadership, culture shifts, etc.) on a continuous timeline, to later summarize and reflect on the findings, and look for emergent patterns. The same exercise can be done in academics however, shifting the focus from an “organisation’s history” to a particular research field or research topic; being “microbiome research through visual analysis.” In the interest of this study, development of microbiome research through visual analysis was broken down in three separate questions: 1) How did the interest (coverage) of microbiome aspects develop over time in the collection of reviewed analysis tools?, 2) How did the methods used to capture these microbiome aspects develop or change over time?, 3) How did the use of platforms to host these visual analysis tools change over time? Like in the Card Sort game, the answers to these questions were provided based on frequency of occurrence in the literature (i.e., if a certain tool offers Shannon diversity to capture within sample diversity, it is listed on the timeline of methods used to capture within sample or alpha diversity). Hence, multiple timelines were created; one containing the aspect coverage, one representing the used platforms, and one for each aspect individually to show the methodological development and visual representations over time. An example of such an exercise can be found in the Supplementary Figure S2.

### 2.3 Workshops

To further explore and dive deeper into the results captured by the individual literature review analysis, similar exercises were done within a focus group of domain experts related to the microbiome. As experts in a complex research field may sometimes experience difficulties expressing their research objectives and needs due to the inherently exploratory nature of the analysis, data and its uncertainties, literature suggests the use of domain characterization exercises to facilitate communication and information sharing within interdisciplinary groups of experts (Munzner, 2009; Panagiotidou et al., 2020). The expert groups were drawn from three different research domains (biologists, statisticians, and visualization experts), to obtain diverge insights coming from different perspectives. In total, 2 workshops were organized. The first workshop included 4 participants, among which 1 microbiologist, 2 bio-statisticians and 1 visualization expert. The second workshop included 1 microbiologist, 3 bio-statisticians and 1 visualization expert. The same visualization expert was present in both meetings, whereas all other participants within the focus group changed. Due to COVID-19, the second workshop had to be done virtually using the online collaborative whiteboard platform Miro (miro.com). The first meeting could be done in person. The meetings took between 1 h and 30 min and 2 h, using an informal “game” structured setting. An informal setting was chosen to create an open and friendly environment to establish collegiality and trust across participants (Knoll et al., 2020). The workshops were conducted in three phases; 1) introduction, 2) Post-Up, and 3) Card Sorting.

At the start of the workshop, goals and guidelines for the participants were communicated, followed by a short introduction round and warm up exercise. According to Kerzner et al. (2019), the latter encourages idea generation and self expression and consequently advances in agency.

The second phase of the workshop aimed at generating ideas. During this phase a *post-up* game (Gray et al., 2010) was played to support brainstorming. The idea of this game is to start with a question on which the group of participants will search answers to. The question should be written down somewhere (e.g., on a whiteboard) such that participants can consult it at any time. The brainstorm is done individually, and answers should be written down on separate sticky notes. Answers can then be shared and sorted underneath the question and briefly presented toward the group after a set amount of time; being 2 min within our setting. The intend of this game was to compare the experts’ knowledge and needs to what is currently available in the microbiome visualization tools. In this set-up, five questions were asked:

• Q1: Conceptually, what information/knowledge can we gain or would we like to obtain from doing microbiome research? For example: influence of food on obesity, how drugs change the gut microbiome, etc.
• Q2: Which data is required or relevant to obtain this knowledge? For example: location, time, etc.?
• Q3: To answer questions of Q1: which specific aspects can be retrieved from the OTU/ASV abundance table? e.g., taxonomic abundance, most present taxonomies in collected samples.
• Q4: Given the aspects you wrote down before, can you think about methods needed and or used (statistically, visually) to obtain this information.
• Q5: When you think about your own research, I’m interested in the platforms, tools, packages you have used, or are using currently to analyze the microbiome. Can you list these up?

**FIGURE 1:**
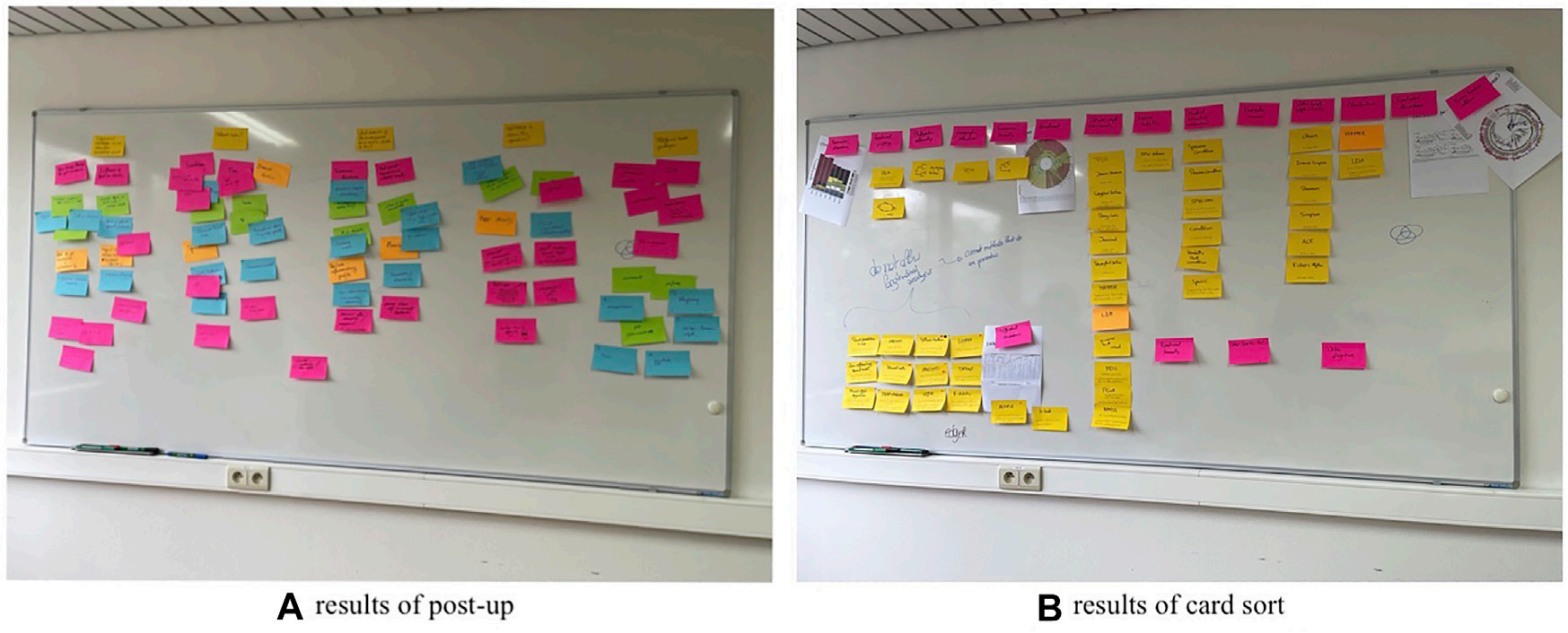
Phase two and three of the workshops; **(A)** a post up brainstorm sessions in which participants were asked to provide their knowledge on 5 microbiome analysis related questions, and **(B)** a closed card sorting to provide their experts opinion on currently used methods. The actual results of the post up session can be found in supplementary material (Supplementary Tables S1, S5).

An image of the workshop environment at the end of this phase is shown in Figure 1A, and the list of provided answers can be found in the supplementary materials (Supplementary Tables S1, S5).

Phase three of the workshop included the same closed card sort game as performed in the individual reviewing process. The same cards and categories were provided to the expert panel and the objective of the game was the same, only this time sorting was based on experts’ knowledge rather than frequency of occurrence in literature; allowing to easily identify discrepancies between experts opinions and literature. Therefore only one card was provided for each statistical method, visualization algorithm or visual design this time, regardless frequency of use. Still, participants were free to duplicate cards. All categories were briefly explained before the start of the game. Each card also contained concise description of the method. Based on this information, participants were asked to sort the card under the categories they believed it could be used for. Furthermore, participants were also allowed to create additional cards and categories containing methods and aspects not covered in the tools. At the end, participants were asked to conduct a value mapping through dot voting (Gray et al., 2010) on the cards that had been sorted. Statistical methods, visualization algorithms and visual designs that experts believed were still informative and insightful obtained a dot, providing an indication of the ones that are still accurate and useful in microbiome research, which could result in interesting discussions. An image of the workshop environment at the end of this exercise is presented in Figure 1B.

Important with these type of exercises is to promote open communication among participants to obtain as much context and background knowledge as possible, and acknowledge expertise from all participants to gain as much input as possible (Kerzner et al., 2019). The workshops were recorded for later reference during analysis with permission of the participants.

## 3 Results

### 3.1 Research Objectives

Based on the literature and the answers to Q1 of the post up game (i.e., Conceptually, what information/knowledge can we gain or would we like to obtain from doing microbiome research?), several objectives were identified in which microbiome research can play a role. The responses of the experts on the question “what information or knowledge can or could be obtained from microbiome research?” could be categorized in 5 major objectives. The first, and most prominent research objective listed by the experts is the association between the microbiome and diseases, among which obesity and multiple sclerosis. All experts believed there is a role to play for the microbiome in disease treatment. Currently, drugs are used for disease treatment, but more research is required on whether they directly affect the disease or whether the effect is mediated through the gut microbiome. If the latter is true, drug alternatives such as a specific diet or fecal therapy could play a prominent role. The second topic of interest that came forward during the discussions was the effect of environmental and personal conditions on microbiome composition. These include seasonal changes (e.g., sunlight), geographical location, past diseases, diet, etc. The third topic listed during the discussions was the role for the microbiome in agriculture, specifically its effect on plant growth/production. Next, psychological associations were listed as a topic of interest. Literature has shown that a link between the gut microbiome and psychological diseases (e.g., depression) exists (Dash et al., 2015; Winter et al., 2018), but does the gut microbiome composition also alter our mood? Lastly, the experts expressed interest in the role of the microbiome in areas such as crime investigation. This could be in revealing social contact patterns based on similar microbiome compositions, using the skin microbiome to see who had physical contact with whom, but also with certain objects or animals, etc. A commonality between all the topics listed above is that they all rely on finding the association between the microbiome (s) and other parameters, and more interestingly (if possible) in revealing causal relationships.

### 3.2 Data Requirements

Qualitative data is needed to provide accurate answers to these research objectives. Based on the answers and discussion on Q2 of the post up game (i.e., Which data is required or relevant to obtain this knowledge?), a general outline of “qualitative data collection in microbiome research” could be established. Besides the need of qualitative genome sequencing, samples should be accompanied by a set of metadata containing additional information about the host and its environment, the (clinical) study, and the sample collection. Specifically, baseline characteristics of the host should be captured (e.g., if human: age, gender, geographic location, etc.); environment information from the host (e.g., exposure to certain chemicals, passive smoker, diet, etc.); clinical information from both the host and the clinical trial study; and information about sample collection (e.g., timestamp, sample location within the host). Furthermore, to obtain metabolic information, accurate databases are required for functional profiling. A full list of the answers provided to Q2 can be found in the supplementary material (Supplementary Table S2).

### 3.3 Methods and Algorithms in Microbiome Research

To analyse this data and investigate previously listed research objectives, an interplay between statistical methods, algorithmic visualizations and (interactive) visual representations are required. These allow us to reveal certain aspects of the microbiome which accordingly permit us to provide answers to these research objectives.

#### 3.3.1 A Changing Research Landscape

**FIGURE 2:**
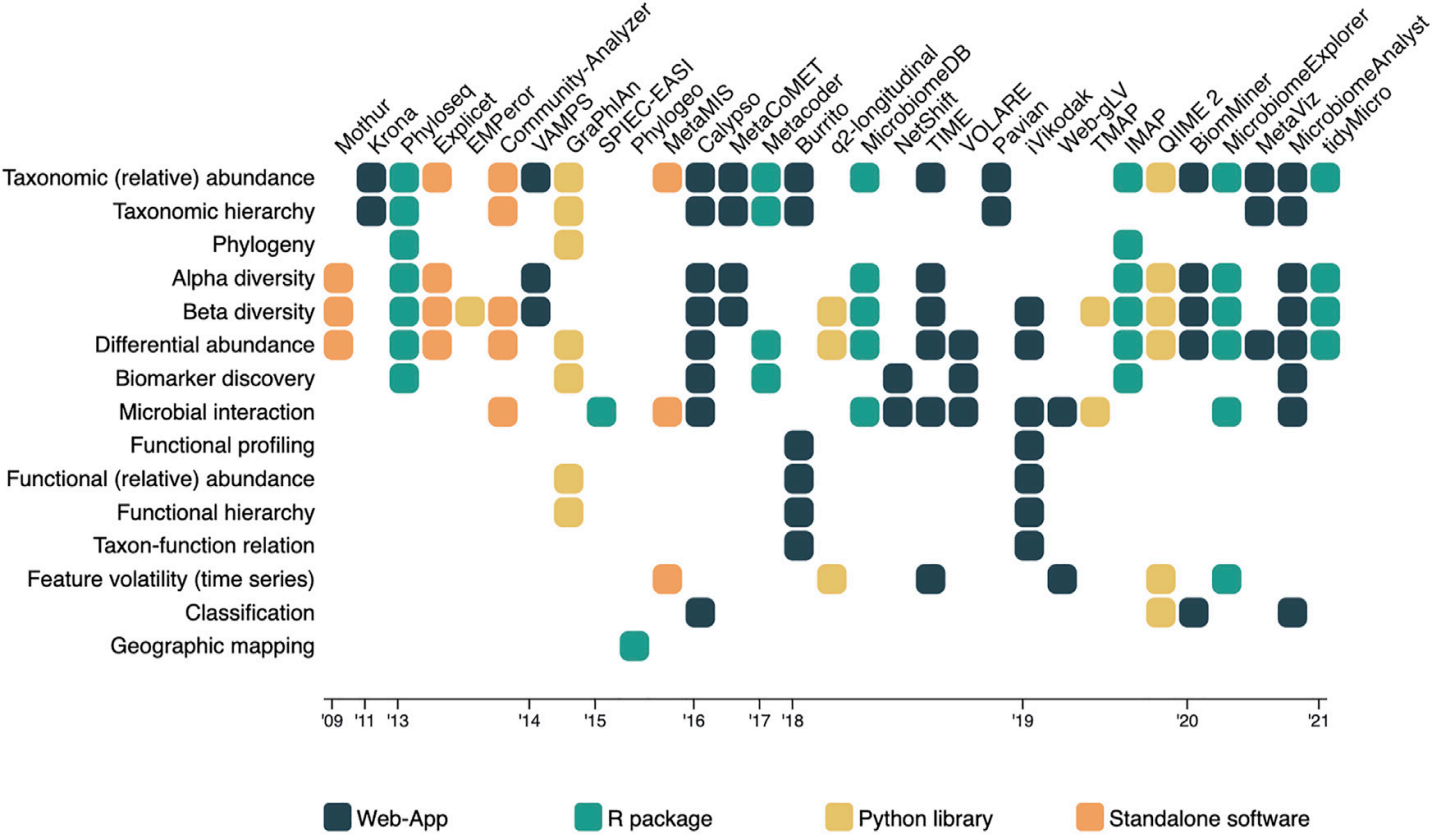
A matrix overview of the tools and algorithms included in the literature review, in which the tools and algorithms are represented in the columns, and the microbiome aspects they measure and present listed as rows. Cells indicate the coverage of an aspect by the corresponding tool, and are colored based on the platform they were hosted on.

The rapid development of these methods and algorithms in microbiome research is clearly visible in the literature. The first visualization oriented microbiome analysis tools only covered the visualization of taxonomic abundance and relationships (Ondov et al., 2011), and the exploration of within- and between-sample diversity (Schloss et al., 2009). Not many years later, tools started to implement methods to test for statistical differences between samples in terms of abundance (differential abundance analysis), and statistical differences between cohorts or populations that can be related to a particular (disease) condition (biomarker discovery) (McMurdie and Holmes, 2013; Robertson et al., 2013; Weiss et al., 2017). During the same period, the first tools allowing for visual exploration of microbial interactions and associations became available as well (Kuntal et al., 2013), used to get an idea about which microbes tend to co-occur with each other. Meta data also became more important in the analysis of diversity between microbiome samples. It is more and more explored together with the on taxonomic abundance based diversity scores (Vázquez-Baeza et al., 2013; Zakrzewski et al., 2017; Liao et al., 2019). In the latest years, major developments occurred; enrichment analysis found its way into the microbiome visual analysis tools (Kuntal et al., 2016; Chong et al., 2020), researchers are now able to visualize and investigate taxon-function relationships (McNally et al., 2018), and tools were developed for longitudinal studies including feature volatility and time series analysis (Baksi et al., 2018; Bokulich et al., 2018). The latest development in the field was the introduction of machine learning (ML) classifiers (Chong et al., 2020; Shamsaddini et al., 2020). Regardless of the fast development and progression in microbiome research and its visual analysis tools, all types of analyses and aspects of the microbiome have remained relevant for exploration. This observation was made based on the fact that older methods (e.g., diversity indices) are still implemented in newer published tools (Carpenter et al., 2021), and confirmed by the expert panel focus group discussions. Figure 2 provides an overview of which microbiome aspects are currently covered by which tool.

#### 3.3.2 Aspects

In Q3 of the post up game, we asked our participants to list all aspects that could be extracted from an OTU/ASV abundance table in order to answer the research questions provided on Q1. A wide variety of features were provided and could be categorized into 4 major research interests: 1) exploratory analysis of baseline characteristics such as (relative) abundance, variability, diversity and richness, 2) statistical effect modelling to obtain effect sizes and p-values, and identify differences taxa abundance and discover biomarkers, 3) interaction models to reveal the interrelationship between taxa, and 4) functional analysis of taxa. In the following we discuss the aspects that were found to be extracted in literature, supplemented with important findings that came up during the workshops (answers to Q4 and card sort) and review process.

##### (Relative) Abundance

Perhaps the most important thing in microbiome research is the ability to look into the (relative) abundance of taxa within and across samples. It provides a first impression of which taxa (functions) are most prominent within a sample, group or population, and can guide us into certain directions of interests. Due to the compositional structure of the data in microbiome research, one tends to prefer looking into relative abundances rather than absolute abundances. An exploration of the (relative) abundances involves no complex statistical modelling, and can be easily done by means of some descriptive statistics and a visual representation of the data.

**FIGURE 3:**
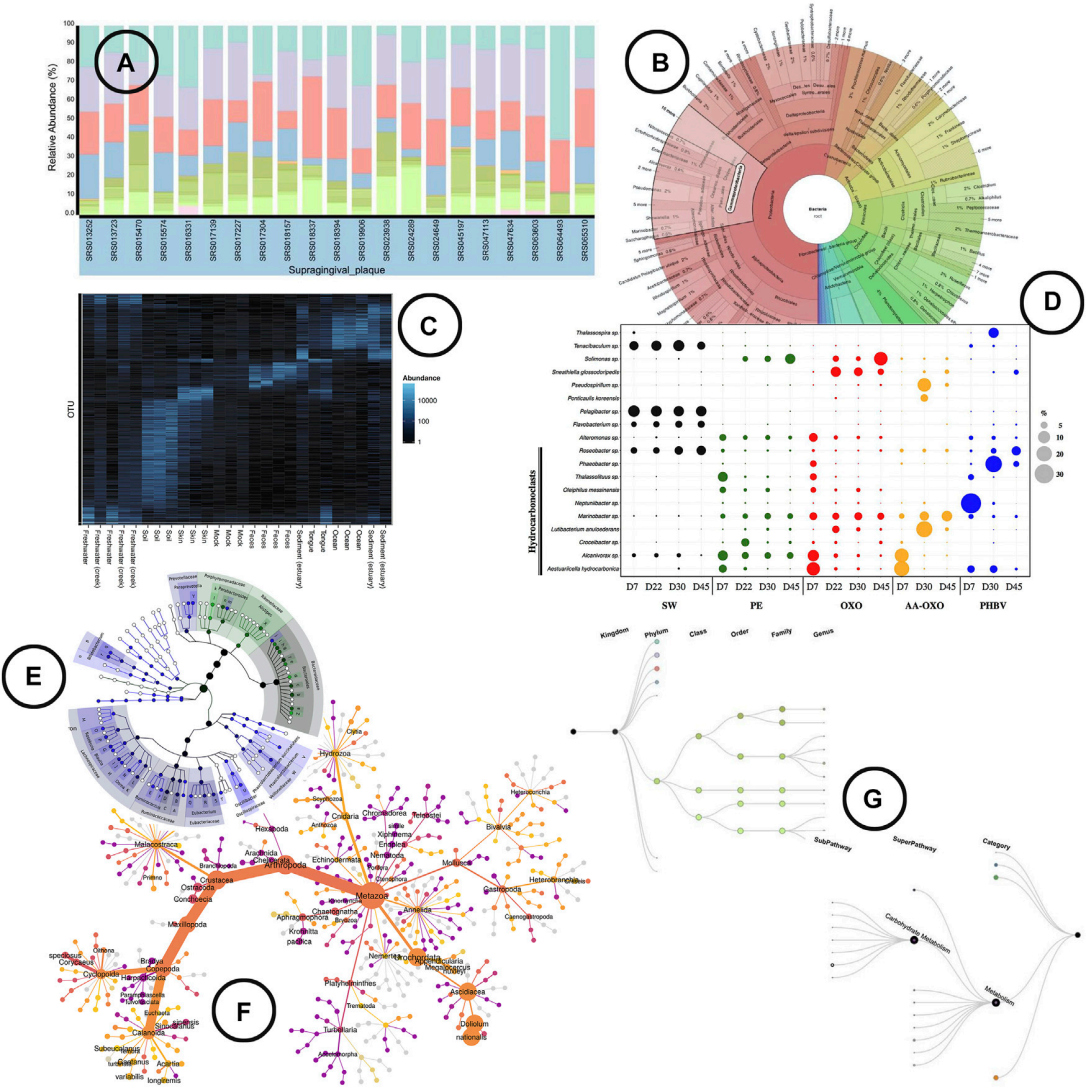
An overview of the visual encodings used to display (relative) abundance and hierarchical/relational structures; **(A)** relative abundance displayed by means of a stacked bar chart in BURRITO (McNally et al., 2018), **(B)** a krona sunburst chart showing the taxonomic hierarchy of the observed bacteria and their relative abundance (Ondov et al., 2011), **(C)** OTU abundance visualized as a heatmap using Phyloseq (McMurdie and Holmes, 2013), **(D)** relative abundance of OTUs represented in a bubble plot (Dussud et al., 2018), **(E)** GraPhlAn, a tree based visualization tool that allows to add visual annotations (Asnicar et al., 2015), **(F)** a “heat tree” visualization showing the taxonomic hierarchy within its tree structure and OTU abundance using node width (Foster et al., 2017), **(G)** taxa and function hierarchy displayed within tree structures in BURRITO with node width representing abundance (McNally et al., 2018).

**Visualization**—Stacked or regular bar-charts seem to be the most prevalent visual encodings to do so, although they are limited in the number of species (functions) they can visualize for the chart to still be readable (Knaflic, 2015). Heatmaps are a frequently used alternative that allow us to visualize all species (functions) at once. The use of color intensity as a channel in heatmaps on the other hand makes the comparison in terms of relative abundance a bit harder than using length (bars) (Munzner, 2014). Nonetheless, does the use of color allows us to easily include (relative) abundance visualization in other microbiome aspect oriented visualizations [e.g., alongside taxonomic classification (Ondov et al., 2011)]. Other alternative visual encodings found in literature include the use of angle [e.g., sunburst chart (Ondov et al., 2011)] and area [e.g., bubble plot (Dussud et al., 2018)] to display (relative) abundance. An overview of how visualization is been used to represent (relative) abundance in literature is shown in Figure 3.

##### Hierarchical/Relational Structures

Microbiome analysis can be done up to different levels depending on the interest of the study, and the sequencing process used to sample the data. In general, sequencing up to a deeper level provides more detailed information. On the other hand, does it bring more problems into the analysis due to sparseness. Most statistical models are not suited to handle many zero counts in the data (Knight et al., 2018).

**Visualization**—In the analysis of microbiome samples, it can be interesting to visually represent the hierarchical level of the taxonomies (domain, kingdom, phylum, class, order, family, genus, species), hierarchical level of the functions (category e.g., metabolism, superpathway e.g., carbohydrate metabolism, subpathway e.g., glycolysis), or even the phylogenetic relationship of the species. Tree structures (including radial trees, cladograms, etc.) are the typical visual encodings used, and are basically the only visual encoding found in literature (Figures 3B,E–G).

##### Within Sample (Alpha) Diversity

Alpha diversity provides an idea of the diversity of species within a particular sample. This metric is often used as a biomarker (Prehn-Kristensen et al., 2018) in disease association studies, but also as a check of sample quality (Schloss et al., 2009).

**Analysis**—Looking into alpha diversity calculations and visual representations, no clear evolution could be found. Many different options exist and are used, but no uniform standard has emerged yet. Typically, alpha diversity metrics can be distinguished into two types: richness- and evenness-measures; *Chao1* being the most used richness metric, and *Shannon* the most used evenness metric. A full list of alpha diversity measures is provided by Hagerty et al. (2020). The authors advocate for the use of a composite metric based on exploratory factor analysis (EFA), taking into account both richness and evenness metrics unified in one.

**FIGURE 4:**
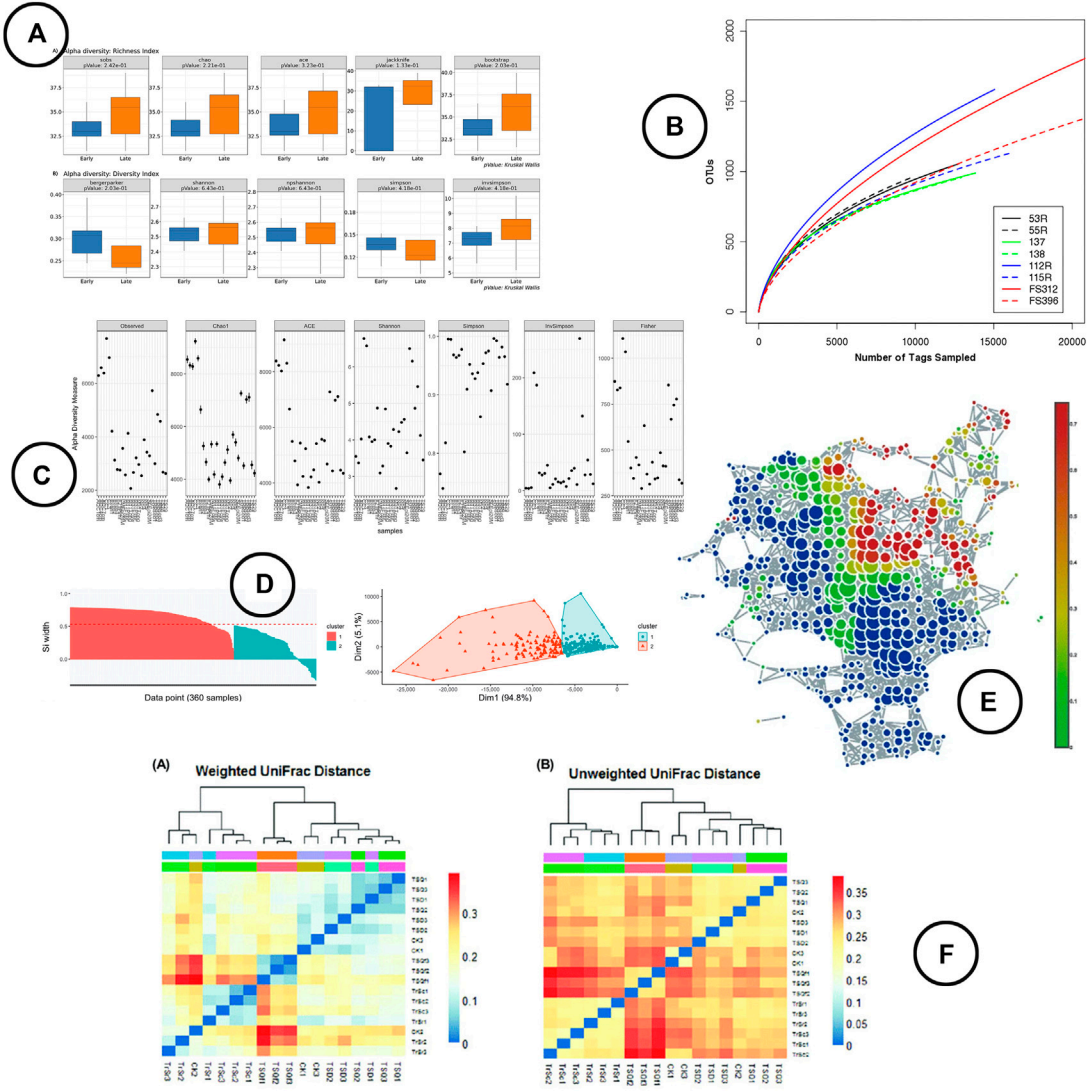
An overview of the visual encodings used to display within (alpha) and between (beta) diversity; **(A)** alpha diversity metrics compared between groups by means of box-plots in BiomMiner (Shamsaddini et al., 2020), **(B)** rarefaction curve showing the number of OTUs by sequence size in Mothur (Schloss et al., 2009), **(C)** alpha diversity metrics visualized using scatter plots in Phyloseq (McMurdie and Holmes, 2013), **(D)** beta diversity visualized using ordination in iMAP (Buza et al., 2019), **(E)** a node-link diagram produced using TDA in TMAP to display beta diversity (Liao et al., 2019), **(F)** heatmap visualizations showing beta diversity distance matrices (Lei et al., 2017).

**Visualization**—box-plots are widely used to display alpha diversity if the objective is to make a comparison between sample cohorts. Line-charts (rarefaction curves) and scatter-plots tend to be used more frequently when visualizing the metrics across samples; the rarefaction curve presenting the (predicted) sample richness by sequence size, often used for re-sampling. Venn diagrams are used to display which part of the microbial taxa are present in multiple samples in relation to the total diversity within those samples. An overview of the visuals used to represent the within sample diversity is given in Figure 4A–C.

##### Between Sample (Beta) Diversity

Beta diversity represents the diversity of species across samples, commonly used to find clusters of similar samples. Typically, this feature is calculated in the exploratory analysis, as it provides a first impression on which taxa are important to distinguish samples, but also on how microbial compositions are related to environmental and personal meta data. With regard to the research objectives listed above, social contact networks could for instance be revealed based on similar microbiome compositions of the skin.

**Analysis**—Beta diversity is expressed as a distance matrix calculation on relative OTU abundance, which serves as an input for visual exploration of sample divergence and similarity. Often occurring distance metrics are: (*weighted*) *UniFrac*, *Jaccard*, *Bray-Curtis* and *Jenson-Shannon* (Oliveira et al., 2018; Chong et al., 2020; Shamsaddini et al., 2020). An important note however is that none of these measures account for the compositionality of the data. Compositional replacements for these distance metrics have been developed; *philr* (Silverman et al., 2017) as a replacement for (weighted) UniFrac, and *Aitchison distance* (Aitchison et al., 2000) for Jensen-Shannon divergence and the Bray-Curtis dissimilarity metrics. Nevertheless, implementation is lacking in the microbiome visual analysis tools.

From 2019 onward, a new trend seemed to develop, which is to test for statistical significance of the between-sample differences (ordination measures). Statistical tests used for this include AMOVA, HOMOVA, ANOSIM, PERMANOVA, PERMDISP, and LIBSSHUFF (Buza et al., 2019; Chong et al., 2020; Shamsaddini et al., 2020). One important recent development is that ordination analysis techniques can be performed on sample functional potentials rather than their taxonomic proportions (Nagpal et al., 2019).

**Visualization**—The visual representation of beta diversity can be either directly through heatmaps of the distance matrix (Lei et al., 2017), through ordination based methods (e.g., PCoA, NMDS) which present the samples in a 2 or 3 dimensional space using dimensionality reduction techniques (Vázquez-Baeza et al., 2013; Wang et al., 2016; Bolyen et al., 2019), or by means of network visualizations based on topological data analysis (TDA) (Liao et al., 2019) or cut-off based edges (McMurdie and Holmes, 2013). Note that because of the compositional ignorance in the commonly used distance metrics, samples will be almost exclusively discriminated based on the features that are most abundant realtive to the others features and not on the most variable ones between samples. Therefore, sample location could vary a lot in ordination plots when different features are included or excluded (Gloor et al., 2017). An example of the visual encodings listed above is shown in Figure 4D–F.

##### Differential Abundance

With differential abundance analysis, OTUs that differ significantly between samples, cohorts or populations are identified using statistical hypothesis testing. In doing so, taxa can be related to a certain response (e.g., disease state, growth process).

**Analysis**—The search for the ideal analysis method for differential abundance is still ongoing (Hawinkel et al., 2019). To date, it has been proven that distributional assumptions do not hold for the majority of the taxa, leading to poor performance of parametric models (Hawinkel et al., 2020). The problem with non parametric rank alternatives such as Wilcoxon is that they are typically less powerful in comparison to parametric tests due to their vulnerability to ties in the data (Jonsson et al., 2016). Custom methods have been developed to test on significant differences between microbiome data, taking the compositionality of the data into account (e.g., ANCOM, ALDEx2) (Gloor et al., 2017). In comparison to the complete lack of awareness in Beta diversity analyses, differential relative abundance analysis methods relying on these compositional assumptions are present in some visual analysis tools (Zakrzewski et al., 2017). Yet, another possible solution lies in semiparametric models, such as Probabilistic Index Models (PIM) (Thas et al., 2012). These are based on rank tests (non parametric), but allow for estimates of effect sizes and inclusion of continuous covariates. So far, they haven’t been introduced in microbiome visual analysis tools in a significant way. An important note that came up during one of the workshops, is that the methods used in visual analysis tools are all limited to cross sectional analysis. To the awareness of the expert panel, methods that do allow differential abundance testing in longitudinal studies are sparse, and mostly parametric. Besides, with the currently offered methods, conclusions can only be drawn about associations between taxa and meta data identifying sample cohorts, whereas inference on causality would be of major interest. In recent years, several methods have been proposed relying on structural equation models to reveal the direct and mediation effect of the microbiome on a certain response (Sohn and Li, 2019; Wang et al., 2020). These however cannot be found in the current visual analysis tools. Nonetheless, these methods suffer from validity issues (Vanderweele and Vansteelandt, 2009).

**FIGURE 5:**
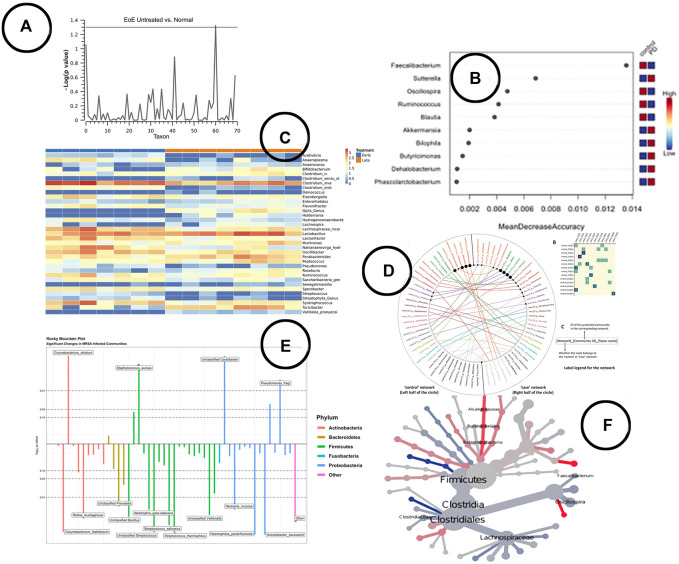
An overview of the visual encodings used to display differential abundant taxa and identified biomarkers; **(A)** Manhattan plot showing statistically significant differential abundant taxa (Harris et al., 2015), **(B)** a visual presentation of the most significant taxa (potential biomarkers) (Cosma-Grigorov et al., 2020), **(C)** difference in abundance of significant taxa shown in a heatmap in BiomMiner (Shamsaddini et al., 2020), (**D**) “community shuffling plot” showing the changes in microbial interactions between clinical groups in Netshift (Kuntal et al., 2019a), **(E)** rocky mountain plot indicating differential abundant taxa in tidyMicro (Carpenter et al., 2021), **(F)** a heat tree visualization showing significantly different taxa between disease and control group (Cosma-Grigorov et al., 2020).

**Visualization**—To visualize statistical significance, several visual encodings have been used; ranging from simple heatmaps and box-plots, to more complex visuals like the Manhattan plot (Harris et al., 2015), rocky mountain plot (Carpenter et al., 2021), volcano plot (Shamsaddini et al., 2020) or heat tree (Foster et al., 2017). An overview of some of the visualizations found in literature is given in Figures 5A,C,E,F.

##### 3.3.2.5 Biomarker Discovery

Biomarker discovery focuses on finding specific parameters or indicators, called biomarkers, that can be related (assigned) to a particular condition (disease).

**Analysis**—When it comes to biomarker discovery, two schools of thought can be distinguished: one using predictive models such as machine learning classifiers, and the other based on hypothesis testing. Among the predictive models, LEfSE (Swenson and Swenson, 2014) is by far the most offered method in the visual analysis tools, followed by some other machine learning algorithms. Methods based on hypothesis testing include methods for statistical difference testing between groups (both parametric and non-parametric). Similar to differential abundance testing, models for clinical studies that take into account the effect of an intervention on both the response (immune response) and biomarkers can be of interest as well. The primary difference however is that their focus is merely on association rather than causal relationships. To the best of our knowledge, there are only two tools that test for association between biomarkers (microbiome taxa compositions) and clinical response variables: NetShift using an algorithmic visualization (Kuntal et al., 2019a), and PhyloSeq using supervised methods (i.e., canonical correspondence analysis, discriminant correspondence analysis, sparse linear discriminant analysis, etc.) (McMurdie and Holmes, 2013). The authors of IVikodak listed the quantification of association between specific sets of bacteria with disease state as a planned future enhancement (Nagpal et al., 2019). None of them however allow for longitudinal analysis, taking into account the effect of an intervention on both the biomarkers and disease response.

**Visualization**—A wide variety of visual encodings have been used to represent the result of biomarker discovery analysis; ranging from simple heatmaps and bar charts, to more complex visuals like the volcano plot (Shamsaddini et al., 2020) and heat trees (Foster et al., 2017). An ongoing search noted by one of the experts in the focus group discussions is on how to visually represent the results of clinical longitudinal intervention studies: how do microbial composition and clinical response variables change over time given a particular intervention. In Figures 5B,D,F, some of the visualizations used in the visual analysis tools are shown.

##### Classification

Classification is used to classify samples in predefined groups based on their microbial composition. It provides information on the most important features (taxa) within sample cohorts, and is therefore often returning as a method for biomarker identification as well.

**Analysis**—Classification methods are fairly new in microbiome research, as only the more recently developed visual analysis tools cover these methods (Chong et al., 2020; Shamsaddini et al., 2020). Machine learning algorithms such as random forest classifiers or support vector machines are typically used for this type of analysis.

**FIGURE 6:**
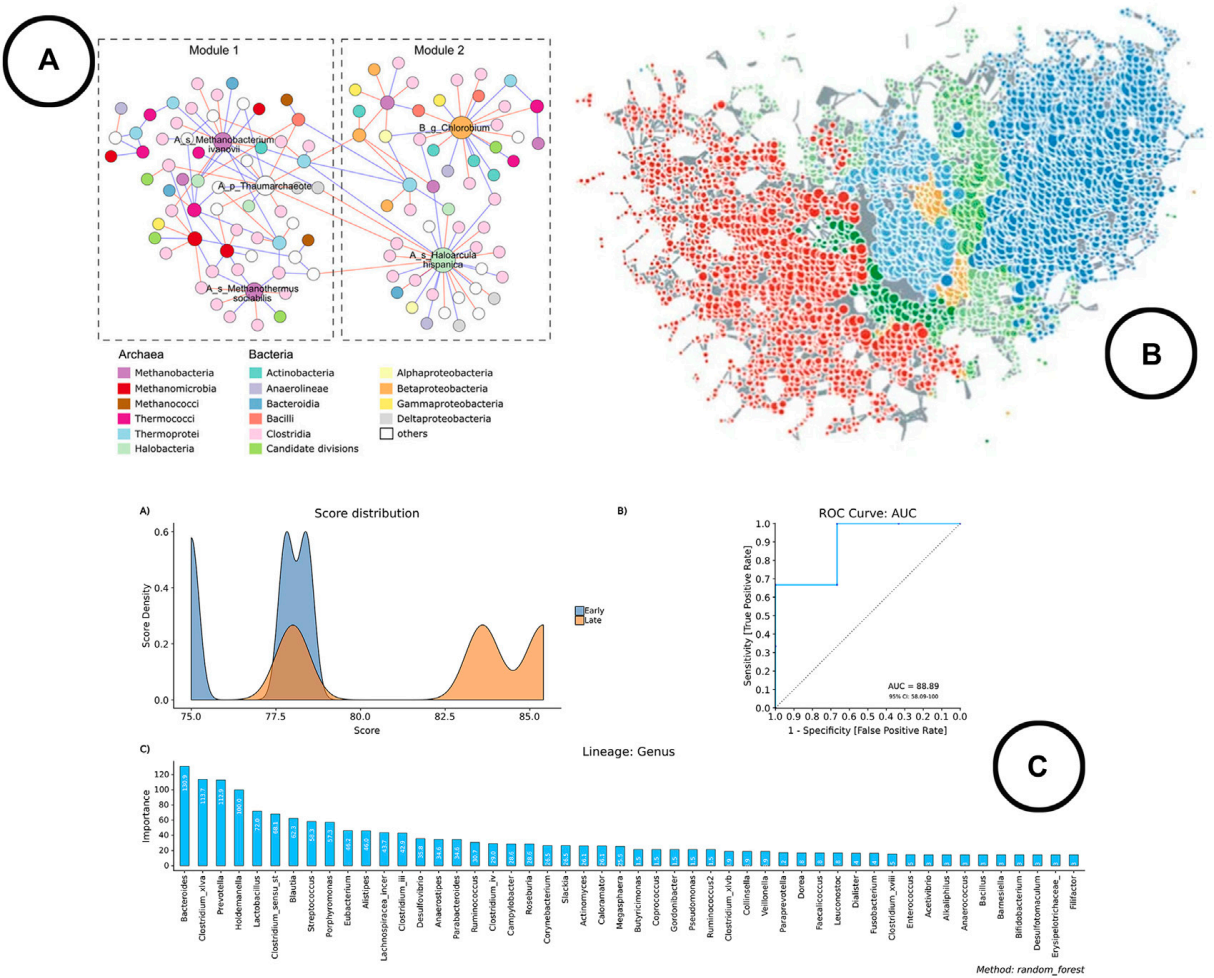
An overview of the visual encodings used to display classification method results and interactions between taxa; **(A)** taxa interaction network (Wu et al., 2020), **(B)** taxa interaction network visualized using TDA in TMAP (Liao et al., 2019), **(C)** visual representation of the results of the Random Forest classifier in BiomMiner (Shamsaddini et al., 2020).

**Visualization**—Line charts (expressed as ROC curves) are typically used to represent model performance, whereas bar charts are used to display the most important features. An example of how this is been shown in literature is given in Figure 6C.

##### Microbial Interaction

The analysis of microbial interaction is focused on identifying the relationship between species. Different types of relations can exist between microbes: mutualistic, commensal, parasitic and competitive (Faust et al., 2012). The goal is to find a method that reveals all of them at once. Identifying these relationships is important for all research objectives listed above. It provides more context on why certain taxa abundances differ in certain situations, and guides us towards possible causal relationships (e.g., is the drug altering the relative OTU abundance or is it altering its relative abundance through another taxa that contains a specific relationship with the OTU of interest).

**Analysis and Visualization**—Looking at the development of microbial interaction analysis within the microbiome visual analysis tools, new methods have been introduced during recent years, which gives an indication that the use of different methods is still further explored. At the moment, three schools of thought can be distinguished: 1) correlation based methods. Problem however with correlation is that it doesn’t correct for the compositionality of the data, and thus leads to spurious correlations (Gloor et al., 2017). Therefore, methods like *SparCC*, *SPIEC-EASI* and *FastSpar* were developed which result in network visualizations based on cut-off values (Chong et al., 2020). 2) Predator-Prey based methods using (generalized) Lotka Volterra equations to model relationships (Shaw et al., 2016; Kuntal et al., 2019b). 3) Topology based methods using topological data analysis (TDA) to construct the networks (Liao et al., 2019). All of these methods result in a graph visualized as a node-link diagram. Figures 6A,B provides an overview of how networks are used to represent microbial interactions.

##### Functional Profiling

As mentioned above on (*relative*) *abundance*, one could also look into the metabolic functions of microbial populations.

**Analysis**—Depending on the type of sequencing, different programs and methods can be used for functional profiling. Galloway-Peña and Hanson (2020) provide a nice overview including use cases and shortcomings. Using 16S rRNA sequencing, methods such as *PICRUSt* (Langille et al., 2013) and *Tax4Fun* (Aßhauer et al., 2015) allow to predict the gene content potential functionality based on a comparison between relative abundances and the reference genome of the taxa present. An important note of the authors that came up in the expert panel discussions as well is that these however are rough approximations, as they don’t take into account actual protein expressions. Using shotgun and metatranscriptome sequencing approaches, tools such as *MetaGeneMark* (Zhu et al., 2010) and *Glimmer-MG* (Kelley et al., 2012) carry out protein sequence homology based searches against databases of orthologues, enzymes, or protein domains and families for gene identification and annotation. The results could then be used for pathway enrichment analysis.

**FIGURE 7:**
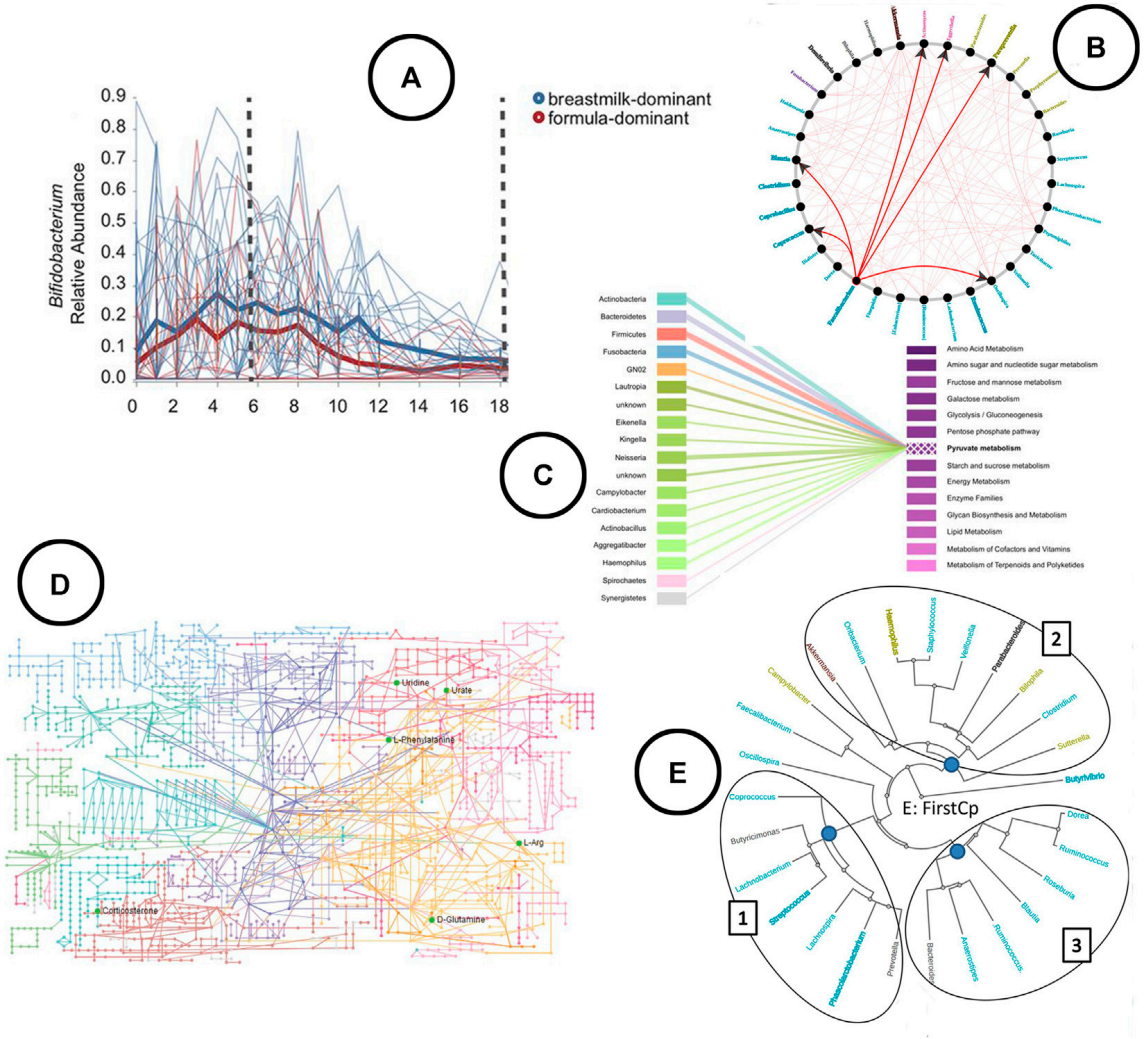
An overview of the visual encodings used to display feature volatility and functional profiling; **(A)** eveloution of relative abundance over time visualized using a linechart in q2-longitudinal (Bokulich et al., 2018), **(B)** associations between taxa based on Granger causality testing represented in a node-link diagram in TIME (Baksi et al., 2018), **(C)** taxa-function relationship displayed using a bipartite graph in BURRITO (McNally et al., 2018), **(D)** KEGG metabolic pathway network (Zhang et al., 2019), **(E)** taxa clustered based on similar trends in time in the web-app TIME (Baksi et al., 2018).

**Visualization**—The link between taxa and functions can be visualized using bipartite graphs (Figure 7C) or interactive stacked bar charts using highlighting, as was done in Burrito (McNally et al., 2018). The result of functional profiling are typically represented in a metabolic pathway network (Figure 7D) (Zhang et al., 2019).

##### Longitudinal Analysis

As mentioned before in the section on differential abundance and repeated in the section on biomarker discovery, to gain a deeper understanding of causal relationships between the microbiome and various sample cohorts (e.g., grouped by disease state), longitudinal studies are required (Secrier and Schneider, 2013). Given the literature reviewed in this study, two tools were found to allow for longitudinal microbiome time series analysis; *TIME* (Baksi et al., 2018), and *q2-longitudinal* (Bokulich et al., 2018), which is an extension on QIIME2.

**Analysis**—In *q2-longitudinal*, linear mixed effect models are used to test for differential abundance. Changes of microbial sample compositions are captured across time using unweighted UniFrac, whereas in *TIME* dynamic time warping distance is used to capture groups of taxa showing similar trends over time. *TIME* identifies causal relationships among taxa using Granger Lasso causality. Stationary taxonomic groups (meaning no inter-microbial competition) are identified using an augmented dickey fuller test.

**Visualization**—Both tools allow for exploration of feature volatility using volatility plots (line charts) (Figure 7A). causal relationships between taxa are displayed using node-link diagrams (Figure 7B); clustering of taxa showing similar trends over time is visualized using a radial tree structure (Figure 7E).

Still, to the best of our knowledge no methods for longitudinal mediation analysis allowing for the identification of causal relationships between intervention, microbiome and response are incorporated yet.

### 3.4 Tools and Platforms

Situating all publications on a timeline (see Figure 2) it becomes clear that initially (2009–2014) tools were mainly made available as standalone downloadable software. Quickly, tools were made available as web applications as well. R and Python are often used to run the analyses on the server side of these web applications (Chong et al., 2020; Reeder et al., 2020), but packages and libraries do also exist to run analyses in the R studio or python programming environments (McMurdie and Holmes, 2013; Buza et al., 2019). The main reason to develop software or web-apps is to remove the constraint of coding, as not all biologist know how to code and learning R or Python might be a bit cumbersome (Huse et al., 2014; Chong et al., 2020). Hence they most often serve as complete analysis pipelines in which microbiome researchers upload their data and can perform different analyses through a point-and-click user interface (Huse et al., 2014). The major problem however with these applications is maintenance. Since standalone software is not open source, updates most often stop when funding stops, as there is nobody who can keep everything up to date besides the developers. A solution to partly alleviate this could be the use of R and Python based server apps like R Shiny (Chang et al., 2015), as was done in Microbiome Explorer (Reeder et al., 2020) or Microbiome Analyst (Chong et al., 2020). Looking into the R packages and Python libraries, three types of packages and libraries can be distinguished: the complete analysis pipeline packages which allow for a thorough and diverse analysis of the microbiome [e.g., Phyloseq (McMurdie and Holmes, 2013), MicrobiomeExplorer (Reeder et al., 2020), IMAP (Buza et al., 2019)], the extensions on these complete packages [e.g. phylogeo (Charlop-Powers and Brady, 2015)], and the computational- or visualization algorithms [e.g. SPIECE-EASI (Kurtz et al., 2015), TMAP (Liao et al., 2019)]. These extensions and algorithms both focus on revealing one particular aspect of the microbiome. During the expert panel group workshops, it became clear that R is primarily used among the participating bio-statisticians. For the creation of a custom visualization, visualization experts make use of web based environments and its according coding languages (HTML, CSS, and JS), and dedicated visualization libraries [D3 (Bostock et al., 2011), p5, etc.].

## 4 Discussion

Based on the expert panel focus group workshops, the main interest in microbiome research is in the identification of associations between the microbiome and host characteristics; be it environmental or health related factors within or among humans, or growth indicators in agriculture. Relevant analysis methods are mainly differential abundance analysis and biomarker discovery. Although these analyses often include metrics like alpha diversity as model parameters, or start from preliminary exploration of the data by looking at the taxonomic compositions and diversity between groups. These methods often include baseline characteristics (e.g., diversity metrics) as model parameters, and proceed from preliminary exploratory analysis of the data.

When it comes to revealing these aspects in the data, several approaches are available. For some aspects the same approach is used exclusively, whereas for others different schools of thought apply. Within sample (alpha) diversity is captured using either richness- or evenness-measures, but a uniform standard is missing (Hagerty et al., 2020). Between sample (beta) diversity is always measured using a distance metric on relative OTU abundance, and stored in a distance matrix. None of the currently implemented distance metrics however accounts for the compositional structure of the data. This compositionality is also one of the major problems for the reliability of statistical hypothesis testing models, which are central in differential abundance testing. Based on the card sorting within the focus group discussions, it became clear that biomarker discovery can rely either on statistical hypothesis testing or predictive modeling. Therefore, many of the methods used in differential abundance testing are found to be used for biomarker discovery as well. Consequently, the same overlap can be found in methods based on predictive modeling which are used for sample classification. A major interest expressed by the expert panel group is the ability to perform causal analysis, which is currently insufficiently developed in differential abundance analysis and biomarker discovery. To do so, the necessity of longitudinal studies and analysis was stressed.

A wide variety of visual encodings exists to represent the data aspects concealed in the OTU abundance tables. Some of these are more unconventional than others, but standard charts (e.g., bar chart, line chart) are most common. Some of them are unconditionally bound to a certain data aspect; hierarchical structures within the data (e.g., taxonomic level) are visualized exclusively using tree structures, connected components are typically used to express relationships (e.g., between taxa, or between functions and taxa), and line charts are most conventional to display evolution over time. Other data aspects on the contrary have been visually represented in many different ways. (Relative) abundance has been visually encoded using channels such as length (e.g., bar chart), color saturation (e.g., heatmap), angle (e.g., Krona), and area (e.g., bubble plot). Based on visualization theory, length would be the most effective channel to display quantitative information such as (relative) abundance (Munzner, 2014), but the use of bar charts however limits the amount of information that can be displayed for it to be still informative. Color saturation on the other hand would be the least effective channel from the ones listed, whereas heatmaps would be the only choice to visually represent the entire data on a static manner. For this reason, heatmaps are also used to visualize beta diversity. It provides a nice overview of the (dis)similarities between samples, although it can become a bit cumbersome to read when the amount of samples is too large. Since the interest is often not limited to the discovery of (dis)similar samples but also in revealing the underlying patterns between samples, ordination based methods are most prevalent in literature. They allow additional data features to be included in the visualization for interpretation, which is not possible using standard heatmaps. The downside of ordination based methods however is that these are limited to a visual representation in a 2 or 3 dimensional space, which might not capture the entire variance to be explained. By displaying the samples using TDA (i.e., node-link diagram), distance between samples is expressed in the edges between the nodes (samples), and therefore no longer relies on the geometric space (Lum et al., 2013). The visualization of the outcomes of statistical models could be as simple as using bar charts and box plots, but have been conducted many times by means of custom visuals as well. In general, the choice depends on the information of interest. If the interest is a list of potential biomarkers (i.e., most important features), a simple bar chart will do and is highly effective according to visualization theory (Munzner, 2014). If the interest is on the effect sizes or any other parameters, more complex and custom visuals are needed.

Here, it is important to also address the issue of visual literacy. In general, the advantage that comes with using standard charts is that everyone can read them. The amount and richness of information that can be shared with them is however limited. On the other hand, custom representations can provide more information in a single graphic but can become hard to read. They should be used with care, by providing the right amount of context needed by the user to understand. An example that emerged during one the workshops was the Rocky Mountain Plot (Figure 5E) used in tidyMicro (Carpenter et al., 2021) to highlight taxa counts correlated with subjects’ age. One could draw conclusions based on the highly correlated taxa counts, but important additional information is missing to draw more accurate conclusions (e.g., variability). Hence, the custom visualization can provide the solution to bring more context to the data analysts, as multiple data aspects can be embedded in the same visual and no longer need to be looked at in isolation [e.g., GraPhlAn (Asnicar et al., 2015)]. In creating these custom visuals, it is imperative that a user-driven design process is used in which visualization expert and domain expert work closely together (Munzner, 2009). Yet, current papers on microbiome visualization and visual analysis mention nothing about the use of design process.

## 5 Limitations

It is sometimes hard to make a clear distinction between tools, as some of them are actually algorithms (e.g., SPIEC-EASI) or visual encodings (e.g., Krona, GraPhlAn) that act and were specifically developed as microbiome visualization tools, but are also embedded as encodings in other tools.

Given the contact constraints added through the COVID-19 pandemic, one of the workshops had be done virtually. As not all participants were familiar with the tools used during this session, additional time was required to familiarize. Nevertheless, both meetings provided a clear overview of some important research topics to cover in microbiome research. The workshop setting was found to be key in structuring discussions, from which interesting information could be obtained such as pointing out current problems and shortcomings. Due to the interdisciplinary composition of the workshops, an additional result was that participants could quickly familiarize themselves in other research domains. We understand that providing examples during the workshops could prime answers into a certain direction. However, due to the interdisciplinary setting of the workshops, we also believe that providing an example helps participants to come to a common understanding of the question asked.
