# Supplementary material for: Exploring the Microbiome Analysis and Visualization Landscape
Source: Front Bioinform. 2021 Dec 2;1:774631. doi: 10.3389/fbinf.2021.774631 (PMC9580862; doi:10.3389/fbinf.2021.774631)
Supplement: Supplementary file 1 [file DataSheet1.pdf]

Supplementary Material

1 SUPPLEMENTARY TABLES AND FIGURES

1.1 Tables

Q1: Conceptually, what information/knowledge can we gain or would we like to obtain from doing microbiome research?
* Influence of food on obesity<br>* How drugs change the gut microbiome<br>* Effect of past diseases (how gut microbiome changes after having had certain disease e.g. salmonella)<br>* Effects on plant growth/production<br>* Role in diseases<br>* Define and find differences in different health and disease statuses.<br>* Effect on health<br>* Influence of food and other factors on microbiome<br>* Microbiome effects on plants (yield more, more productive agriculture)<br>* Impact of bacteria metabolites on treatment efficiency<br>* Role of gut microbiome in autoimmunity (e.g. how microbiome can influence t-cells)<br>* Effect on immune response<br>* Precision medicine<br>* Influence on psychology (mood, depression, . . .)<br>* Who has a dog? (based on skin microbiome)<br>* Changes during day or different seasons<br>* Who lives with whom? (instead of dna, use microbiome to see who was in contact with whom)<br>* Composition of microbiome with varying diet<br>* Microbiome and survival<br>* Composition of microbiome with respect to body combacting diseases<br>* How ”quickly” does diet have effect on microbiome?<br>* Link between diet and longevity?<br>* Use of fecal therapy<br>* Relation between microbiome and MS<br>* How do drugs change the microbiome, and at the same time affect other clinical variables<br>* Diversity and richness

Table S1. Answers provided by the workshop participants to question 1.

Q2: Which data is required or relevant to obtain this knowledge?
* Time<br>* Location<br>* Gender<br>* Age<br>* Exposure data (what did you eat, did you use drugs)<br>* Species (mice, human, other animal)<br>* Disease status (after treatment, before treatment)<br>* Time series (longitudinal sequencing, how dynamic, how quickly does it change)<br>* Which bacteria? + count (absolute)<br>* Diet<br>* Genome<br>* Timestamps<br>* Location of microbiome sample in the body/plant)<br>* Baseline characteristics (e.g. age, gender)<br>* Environmental information<br>* Epidemiological information<br>* Diet<br>* Clinical trial data<br>* Sequencing (amplicon, whole gen.)<br>* Longitudinal information<br>* Treatment and other clinical variables<br>* Disease status/clinical outcome<br>* Metabolic information<br>* Hormonal changes<br>* Microbiomes of contacts<br>* Lifestyle<br>* Health status<br>* Gender<br>* Time and age<br>* Immune response over time

Table S2. Answers provided by the workshop participants to question 2.

Q3: To answer questions of Q1: which specific aspects can be retrieved from the OTU/ASV abundance table?
* Most present taxonomies in collected samples<br>* Taxonomic abundance<br>* Variability of abundances<br>* p-values<br>* Effect sizes (e.g. diff. in relative or absolute abundance between two conditions)<br>* Co-abundance (association measures)<br>* Diversity indices<br>* Absolute/relative abundance (important difference)<br>* Metabolites<br>* Pro/anti-inflammatory profile<br>* Integrated exploration of raw data (of all data listed before)<br>* Variance between different environments/treatments (how different are they)<br>* Variance after re-sampling/removing noisiness, duplicates<br>* Effects of baseline characteristics<br>* Alpha and Beta diversity<br>* Correlation between taxa<br>* Alpha diversity<br>* Biomarker discovery<br>* Relative abundance<br>* Interaction network<br>* Identify pathogens<br>* Metabolite classification<br>* Phylogenetic level<br>* Fat content<br>* Metabolism

Table S3. Answers provided by the workshop participants to question 3.

Q4: Given the aspects you wrote down before, can you think about methods needed and or used (statistically, visually) to obtain this information.
* PCoA (and related ordination methods)<br>* Statistical modeling + differential abundance analysis (DESeq2, EdgeR, ANCOM2)<br>* Venn diagrams (to display; most present taxonomies, compare taxonomic ranks between different samples)<br>* Differential abundance methods (parametric/ non-parametric/ semi-parametric)<br>* (stacked) Bar charts (used to display metabolites)<br>* Dendrograms<br>* Custom visual designs<br>* Parallel coordinates (to display different types of information)<br>* Sample clustering (tsne, UMAP, STAD) $\rightarrow$ scatterplot, node link<br>* Heatmaps (OTU abundance vs timepoints)<br>* Upset (replacement for venn diagram if more than three categories)

Table S4. Answers provided by the workshop participants to question 4.

Q5: When you think about your own research, I'm interested in the platforms, tools, packages you have used, or are using currently to analyze the microbiome. Can you list these up?
* Phyloseq<br>* Microbiome<br>* DESeq2 (diff abundance)<br>* Limma (diff abundance)<br>* EdgeR (diff abundance)<br>* Piur (probabilistic index models)<br>* HMP (Human microbiome project) (data)<br>* phyloseq (R-package)<br>* MicrobiomeHD (datasets + methods)<br>* PICRUST<br>* ggplot2 (for plotting)<br>* D3<br>* HTML/CSS/JS Svelte<br>* Databases (SQL, graph, document store)<br>* Webserver<br>* Pen + paper

Table S5. Answers provided by the workshop participants to question 5.

1.2 Figures

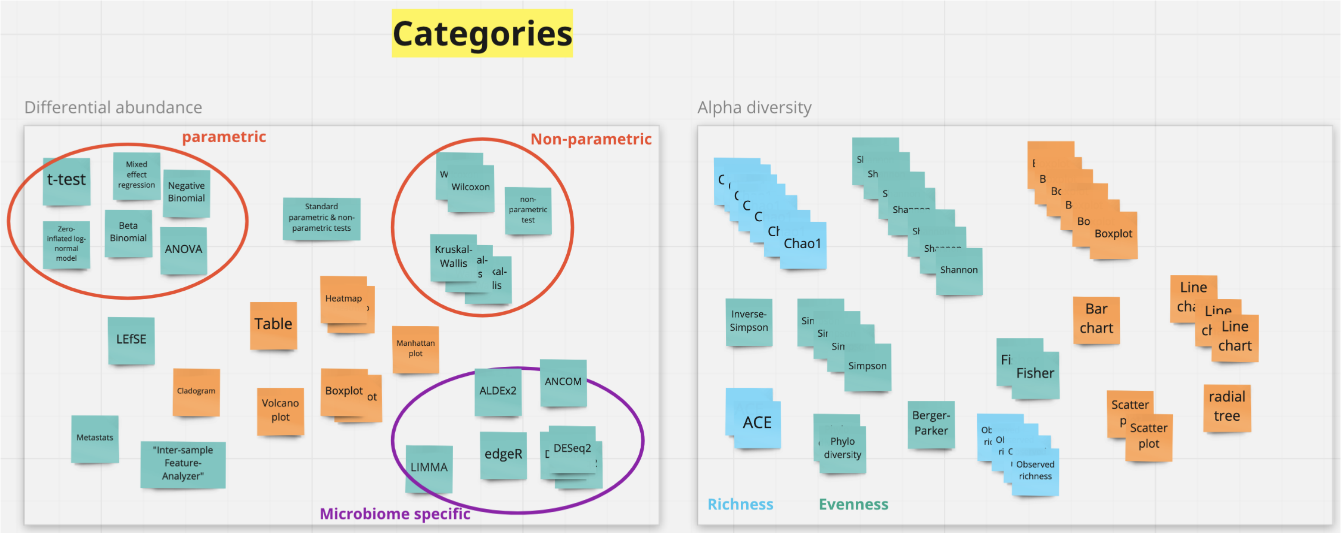

Figure S1. A reproduction of the initial card sort game, displaying how cards are sorted within 2 categories based on frequency of occurrence. The actual exercise were carried out using post its and markers.

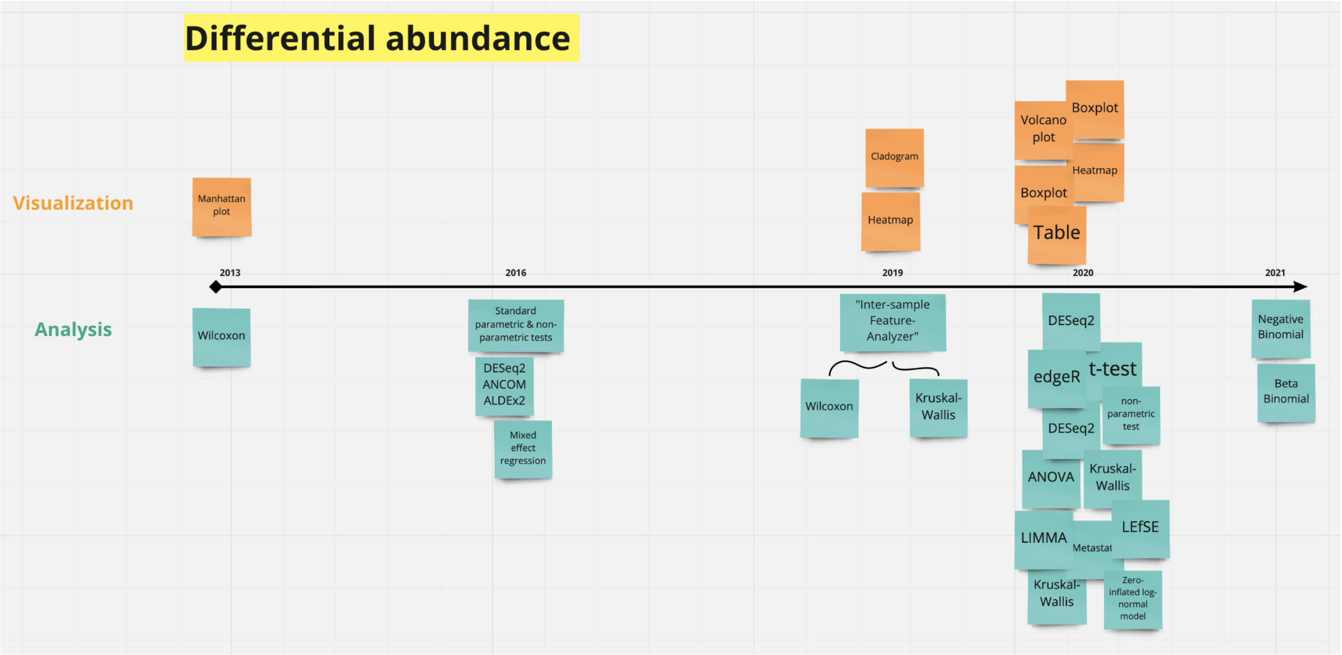

Figure S2. A reproduction of a history map exercise conducted to answer question 2 (i.e. How did the methods used to capture these microbiome aspects develop or change over time?) on differential abundance. The actual exercises were carried out using pen and paper.
